# Real-World Effectiveness of Antiviral Prophylaxis for Preventing Hepatitis B Virus (HBV) Reactivation in Patients Undergoing Immunosuppressive Therapy

**DOI:** 10.3390/v17111436

**Published:** 2025-10-29

**Authors:** İnci Yılmaz Nakir, Bilge Çağlar, Esra Zerdali, Rumeysa Gülistan Karaduman, Filiz Pehlivanoğlu

**Affiliations:** Department of Infectious Diseases and Clinical Microbiology, Haseki Training and Research Hospital, İstanbul 34096, Türkiye; bilge_cglr1907@hotmail.com (B.Ç.); esrayerlikaya@gmail.com (E.Z.); rgkaraduman@gmail.com (R.G.K.); drfiliz@gmail.com (F.P.)

**Keywords:** immunosuppression, entecavir, HBV, reactivation, tenofovir disoproxil fumarate

## Abstract

This study evaluated the characteristics of patients receiving prophylactic antiviral therapy against hepatitis B virus (HBV) infection due to immunosuppressive treatment and assessed the occurrence of HBV reactivation. Between January 2015 and January 2025, 199 adult patients followed in the Infectious Diseases and Clinical Microbiology outpatient clinic who had received prophylactic oral antiviral therapy were retrospectively analyzed. Demographic characteristics, underlying diseases, immunosuppressive treatment history, HBV serological results, biochemical and virological findings, and prophylactic antiviral regimens were recorded. Patients were stratified into low-, moderate-, and high-risk groups according to the American Gastroenterological Association (AGA) 2025 classification. The mean age was 60.4 years; 50.3% of patients were male. Serologically, 26.6% were HBsAg- and anti-HBc-positive, 33.2% showed isolated anti-HBc positivity, and 40.2% had dual anti-HBc/anti-HBs positivity. The risk of HBV reactivation was low in 41.2%, moderate in 22.1%, and high in 36.7% of patients. Prophylaxis consisted of entecavir in 76.4%, tenofovir alafenamide in 13.1%, and tenofovir disoproxil fumarate in 10.5%. HBV reactivation occurred in only one patient, who had discontinued treatment. These findings emphasize the importance of HBV screening and timely prophylactic antiviral therapy in patients undergoing immunosuppression to effectively prevent HBV reactivation.

## 1. Introduction

Hepatitis B virus (HBV) infection is a major global public health issue. It is a leading cause of severe conditions such as chronic hepatitis, liver failure, and hepatocellular carcinoma [1]. The World Health Organization (WHO) reported that in 2022, 254 million people had chronic hepatitis B (CHB). The organization also stated that 1.1 million deaths annually are due to cirrhosis and hepatocellular carcinoma (HCC) caused by HBV infection [2]. The prevalence of Hepatitis B surface antigen (HBsAg) positivity varies by region, with the highest rate of 5.8% found in Africa [2]. In our country, the prevalence of HBsAg positivity has been reported at 4%, indicating a moderately endemic region [1,3]. Additionally, the prevalence of Hepatitis B “core protein” antibody (anti-HBc) positivity in individuals aged 18 and over is reported to be 31% in our country [4].

The natural course of HBV infection depends on the interaction between viral replication and the host’s immune response. Even if a patient achieves clinical and serological recovery and HBsAg disappears from the serum, the HBV genetic material, known as covalently closed circular DNA (cccDNA), can persist in hepatocytes. This situation can lead to HBV reactivation, which may cause viral replication to restart, resulting in hepatitis flare-ups and severe clinical outcomes if immune control mechanisms are disrupted [5,6]. HBV reactivation is common in both HBsAg-positive patients and in HBsAg-negative/anti-HBc-positive patients undergoing immunosuppression. The main cause of this reactivation is immunosuppression induced by treatments [7]. Therefore, it is critically important to screen for HBsAg and anti-HBc in this patient group, especially in regions where HBV is endemic. Close follow-up of these patients is also essential [8,9].

HBV reactivation has been increasingly observed not only in patients with oncologic and hematologic disorders but also among those receiving immunosuppressive therapy for various non-malignant conditions, including rheumatologic, gastrointestinal, neurological, and dermatological diseases [10]. Approaches such as prophylactic or preemptive antiviral treatment should be considered, taking into account the type and duration of immunosuppressive therapy and the patient’s HBV infection status. For prophylactic treatment, antiviral agents such as entecavir (ETV), tenofovir disoproxil fumarate (TDF), or tenofovir alafenamide (TAF) are recommended [3,9,11].

The risk of hepatitis B reactivation due to immunosuppressive therapy varies depending on the patient’s HBV serological status, virological markers, and the specific immunosuppressive agents used. This study aimed to evaluate the characteristics and HBV reactivation status of patients who received prophylactic treatment for hepatitis B infection due to immunosuppressive therapy.

## 2. Materials and Methods

This study was conducted with the approval of the Haseki Training and Research Hospital Ethics Committee (Date: 23 May 2024, Decision No: 37-2024).

This study, designed as a retrospective single-center analysis, evaluated patients who visited our hospital’s Infectious Diseases and Clinical Microbiology outpatient clinic. They were started on prophylactic oral antiviral treatment before beginning immunosuppressive therapy between January 2015 and January 2025. A total of 199 patients were included in the study. They were scheduled for immunosuppressive therapy for autoimmune, malignant, or inflammatory diseases and received antiviral prophylaxis due to positive HBsAg and/or anti-HBc. At baseline, all patients were anti-HBc IgG positive. Among those with isolated anti-HBc IgG positivity or HBsAg positivity, HBeAg and anti-HBe were tested to confirm inactive carrier status. Only anti-HBe–positive (HBeAg-negative) patients were included in the study. Patients who were receiving treatment for chronic hepatitis B or were under 18 years of age were excluded.

The study retrospectively examined data from the hospital information system for all patients included. This data included demographic information (age, gender), underlying diseases, immunosuppressive treatment history, HBV serological results (HBsAg, anti-HBc, anti-HBs), biochemical and virological findings (HBV DNA), and the antiviral agents used for HBV prophylaxis.

Serum HBsAg and anti-HBs levels were measured using a chemiluminescent immunoassay (CLIA) on the Atellica^®^ IM analyzer (Siemens Healthineers, Erlangen, Germany). According to the manufacturer’s instructions, HBsAg positivity was defined as a signal-to-cutoff (S/CO) index ≥ 1.0, while anti-HBs titers ≥ 10 mIU/mL were considered protective.

### 2.1. Immunosuppressive Therapies

- **Rheumatologic diseases**: corticosteroids, azathioprine, methotrexate, cyclosporine, and rituximab; as well as biological agents such as etanercept and golimumab.
- **Hematologic and oncologic diseases**: various chemotherapy protocols containing rituximab, cyclophosphamide, doxorubicin, cisplatin, carboplatin, paclitaxel, gemcitabine, bleomycin, and etoposide; as well as targeted therapies including trastuzumab and bortezomib, and immunotherapies such as nivolumab.
- **Neurological diseases**: ocrelizumab.
- **Gastrointestinal and dermatological diseases**: a combination of azathioprine and corticosteroids (e.g., methylprednisolone) or biological agents such as ustekinumab, secukinumab, and adalimumab (in some cases in combination with methotrexate).

The duration of immunosuppressive therapy varied by underlying disease and clinical indication; however, in most cases, it ranged between 6 months and 1 year.

### 2.2. Definitions

According to the American Gastroenterological Association’s (AGA) 2025 clinical practice guideline for the prevention and treatment of hepatitis B virus (HBV) reactivation in at-risk individuals, HBV reactivation was defined as follows [9].

1. A positive HBsAg test result or an increase in HBV DNA levels of ≥1 log in a patient who was previously HBsAg negative.
2. The reappearance of detectable HBV DNA (≥100 IU/mL) in a patient who is HBsAg negative but anti-HBc positive.
3. The detection of positive HBV DNA along with elevated ALT levels.

Based on the same AGA 2025 guidelines, the risk of HBV reactivation was categorized as high, moderate, or low. This classification was determined by the type of immunosuppressive therapy being used and the patient’s HBV serological status.

### 2.3. Statistical Analysis

For the descriptive statistics of the data, we used mean, standard deviation, median, minimum, maximum, frequency, and percentage values. The distribution of variables was evaluated using the Kolmogorov–Smirnov and Shapiro–Wilk tests.

For the analysis of independent quantitative data that did not follow a normal distribution, we applied the Kruskal–Wallis and Mann–Whitney U tests. The Wilcoxon test was used to compare dependent quantitative data. The Chi-square test was used to analyze independent qualitative data, and Fisher’s exact test was used when the conditions for the Chi-square test were not met.

All analyses were performed using the SPSS 28.0 software.

## 3. Results

**Figure 1:**
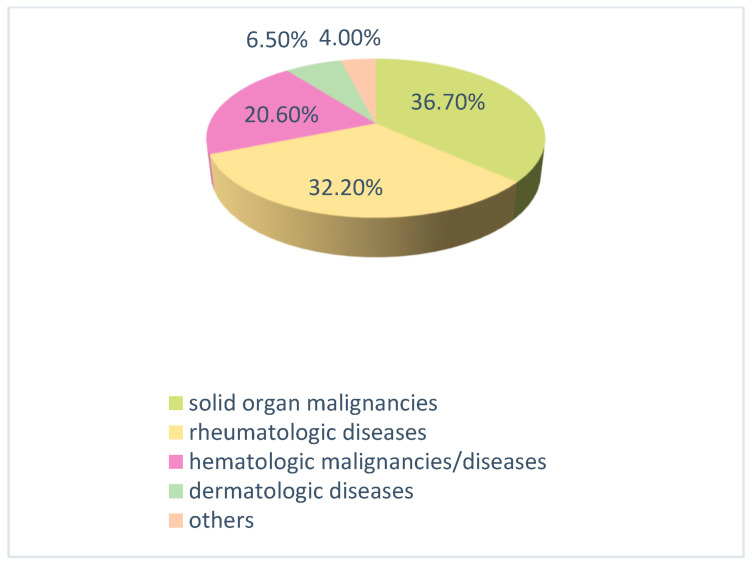
Distribution of patients receiving immunosuppressive therapy according to their primary diseases.

The study included 199 patients, with a mean age of 60.4 years, of whom 100 (50.3%) were male. Regarding the distribution of underlying conditions, the largest group consisted of patients with solid organ malignancies, accounting for 36.7% (*n* = 73). The distribution of other conditions was as follows: rheumatologic diseases 32.2% (*n* = 64), hematologic malignancies/diseases 20.6% (*n* = 41), dermatologic diseases 6.5% (*n* = 13), neurologic diseases 3.5% (*n* = 7), and gastrointestinal diseases 0.5% (*n* = 1) (Figure 1).

Among the patients, 26.6% (*n* = 53) were positive for both HBsAg and anti-HBc, while 33.2% (*n* = 66) had isolated anti-HBc positivity. Both anti-HBc and anti-HBs were positive in 40.2% (*n* = 80) of patients. HBV DNA was undetectable in 162 patients, while 37 patients tested positive. Of these 37 patients, 24 had HBV DNA levels below 2000 IU/mL, and 12 had levels above 2000 IU/mL. All 37 patients who had detectable HBV DNA at baseline achieved undetectable HBV DNA after one year of antiviral prophylaxis. None of the 53 HBsAg-positive patients experienced HBsAg loss during the follow-up period.

The risk of HBV reactivation was assessed as low in 41.2% (*n* = 82) of the patients, moderate in 22.1% (*n* = 44), and high in 36.7% (*n* = 73).

All patients included in the study were started on oral antiviral treatment. Of these patients, 76.4% (*n* = 152) used entecavir (ETV), 13.1% (*n* = 26) used tenofovir alafenamide (TAF), and 10.5% (*n* = 21) used tenofovir disoproxil fumarate (TDF).

Only one patient receiving prophylactic antiviral treatment developed HBV reactivation. This patient, who had undergone an autologous bone marrow transplant for multiple myeloma, was on entecavir prophylaxis. However, the reactivation occurred during a period when the patient had voluntarily discontinued the treatment.

The characteristics of patients by reactivation risk group are summarized in Table 1.

The patients in the study were divided into two groups: those using Entecavir (ETV) and those using Tenofovir (TDF and TAF). The mean age in the ETV group (61.5 ± 11.6) was statistically significantly higher than in the tenofovir group (56.8 ± 14) (*p*: 0.025).

**Figure 2:**
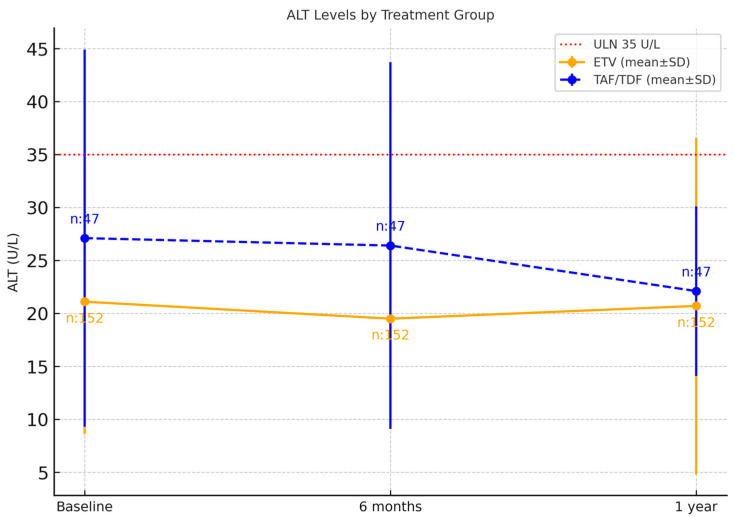
Distribution of ALT levels by drug group throughout treatment. Distribution of ALT levels (mean ± SD) at baseline, 6 months, and 1 year in patients receiving ETV (*n* = 152) and TAF/TDF (*n* = 47). Error bars represent the standard deviation around the mean values. The red dashed horizontal line corresponds to the upper limit of normal (ULN, 35 U/L), providing a reference for interpretation.

At baseline and at 6 months, ALT values were significantly higher in the tenofovir group compared to the entecavir group (*p* < 0.05). At 12 months, although a statistically significant difference was observed (*p* = 0.033), the mean ALT levels of the two groups were very close to each other. In the ETV group, the 6-month ALT value showed a significant decrease from the baseline (*p* < 0.05), but the 1-year ALT value did not show a significant change compared to the baseline (*p* > 0.05). In the Tenofovir group, ALT values at 6 and 12 months did not show a statistically significant change compared to baseline (*p* > 0.05); although a decrease was observed at 12 months, this difference was not statistically significant (Figure 2). Additionally, the percentage of patients with high ALT values at the baseline, 6-month, and 1-year marks was significantly higher in the Tenofovir group compared to the ETV group (*p* < 0.05) (Table 2). There was no significant difference between the two groups in the extent of ALT change from baseline to 6 months or from baseline to 1 year (*p* > 0.05). Among the 31 patients who had elevated ALT at baseline, ALT levels normalized in 58.1% (18/31) at the end of one year, while 41.9% (13/31) continued to have elevated ALT values; of these, 61.5% (8/13) were in the TDF/TAF group and 38.5% (5/13) were in the ETV group. In the TDF/TAF group, elevated liver enzymes at baseline were observed in 29.8% (14/47) of patients, most of whom had autoimmune, rheumatologic, hematologic, or oncologic diseases. At the 6-month follow-up, ALT elevation persisted in 50.0% (7/14) of these patients and at the end of the first year in 42.9% (6/14). Additionally, new ALT elevation at 6 months occurred in 10.6% (5/47) of patients who had normal baseline ALT; all were hematology–oncology cases receiving intensive or rituximab-containing chemotherapy regimens, and in 60.0% (3/5) of them the elevation persisted at one year. None of the patients developed clinical hepatitis or required discontinuation of antiviral therapy.

The characteristics of patients based on the antiviral treatment they received are summarized in Table 2.

## 4. Discussion

Clinical guidelines recommend serological screening for HBV infection in all patients who will receive immunosuppressive therapy and initiating antiviral prophylaxis when necessary [9,11,12]. Patients who have been exposed to HBV and are on immunosuppressive therapy have a 17% to 55% risk of HBV reactivation if they do not receive antiviral prophylaxis [13]. In our study, only one of 199 patients developed HBV reactivation, which occurred after voluntarily discontinuing entecavir therapy following an autologous bone marrow transplant. This underscores that adherence to prophylaxis is crucial for preventing reactivation in patients with prior HBV exposure.

Although our study did not include a control arm, previous studies have clearly demonstrated the preventive effect of antiviral prophylaxis. A meta-analysis of 1312 HBsAg-negative and anti-HBc-positive lymphoma patients receiving rituximab-based chemotherapy reported a 9% HBV reactivation rate among those without prophylaxis [14]. Similarly, the randomized PREBLIN trial found reactivation in 10.7% of untreated patients compared with none in those receiving tenofovir [15]. These findings emphasize that the almost complete absence of reactivation in our cohort reflects the established efficacy of nucleos(t)ide analog prophylaxis.

In a study by Ceylan et al., no reactivation was observed in any of the 105 patients who received prophylactic antiviral treatment, which is consistent with our results [16]. Similarly, in a large Japanese cohort of patients with solid tumors and resolved HBV infection, Kotake et al. reported a low HBV reactivation rate of 2.1% under regular HBV DNA monitoring. Most reactivations occurred in patients with low anti-HBs titers or higher steroid exposure, indicating that HBV reactivation is rare when appropriate prophylaxis and monitoring are applied [17].

The European Association for the Study of the Liver (EASL), the American Gastroenterological Association (AGA), and the American Association for the Study of Liver Diseases (AASLD) all recommend routine HBV serological screening before starting immunosuppressive therapy [9,11,18]. Despite these recommendations, studies show that these screenings are not performed often enough. For instance, the literature notes that only 20.3% of rheumatoid arthritis patients in the U.S. and 24.5% in Taiwan underwent HBV serological testing [10]. Another study conducted in Japan showed that among patients with RA, only 28.23% were tested for HBsAg, 12.52% for anti-HBs, and 14.63% for anti-HBc [19]. These data indicate that HBV screening and protection strategies for patients receiving immunosuppressive therapy need to be strengthened on a global scale.

In patients with solid tumors who do not receive antiviral prophylaxis, the risk of HBV reactivation due to anticancer treatment is 25% for those with chronic HBV and 3% for those with a past HBV infection. In a cohort study of 3465 cancer patients receiving immunosuppressive therapy, 511 had chronic HBV, and 2954 had a past HBV infection. HBV reactivation occurred in only five patients, all with chronic HBV; no reactivation was seen in those with past infection. Of these five cases, three had not received prophylaxis, one was non-adherent, and one had discontinued treatment. This study demonstrates that HBV reactivation can occur in chronic HBV patients who are not on antiviral prophylaxis or are non-adherent to treatment during immunosuppressive therapy [20]. In our study, approximately 60% of the patients were in the moderate or high-risk group for reactivation, yet only one out of 199 patients who received prophylactic antiviral treatment developed reactivation after voluntarily discontinuing their treatment. This result supports the effectiveness of prophylaxis in patients on immunosuppressive therapy while also showing that treatment adherence and compliance must be closely monitored.

Entecavir (ETV) was used by 76.4% of our patients, while 13.1% used TAF and 10.5% used TDF. Due to their high safety profiles with long-term use, ETV, TDF, and TAF are recommended as prophylactic treatments to prevent HBV reactivation in patients scheduled for immunosuppressive therapy [9]. While TDF has a good safety profile, side effects like nephrotoxicity and osteoporosis have been reported [21]. In our study, entecavir was the most commonly used prophylactic antiviral agent, accounting for 76.4% of cases. Similarly, a study by Şahin et al. also found that entecavir was the preferred prophylactic choice for 92.9% of their patients [22]. The decision to start entecavir, TDF, or TAF varies depending on the clinician’s judgment, the patient’s condition, and the underlying disease. In our country, the use of TAF was limited until 2020 due to reimbursement restrictions under the Health Implementation Communiqué. In our study, entecavir was more frequently chosen over TDF, likely because the majority of our patients were elderly with comorbidities. Clinicians may have preferred its high safety profile and lower risk of side effects. Our data revealed that the average age of patients using entecavir was significantly higher than those using tenofovir (*p* = 0.025). This finding suggests that clinicians may be more cautious about the potential side effects of tenofovir (bone and kidney toxicity) in older patients, which could have led to the more frequent preference for entecavir in this group.

In our study, baseline, 6-month, and 1-year ALT levels were significantly higher in the tenofovir group compared to the entecavir group. Patients taking entecavir showed a statistically significant decrease in ALT levels at 6 months (*p* < 0.05), but this difference was not sustained at 1 year. In the tenofovir group, no significant change was observed throughout the follow-up period. Additionally, the percentage of patients with high ALT levels was significantly greater in the tenofovir group at all time points compared to the entecavir group. In the tenofovir group, mean ALT levels decreased from the 6th to the 12th month, yet the number of patients with ALT values above the ULN slightly increased (from 12 to 14). While most patients showed a reduction in ALT, a small number with persistent or newly elevated values accounted for this increase. The heterogeneity of the study population, including frequent use of hepatotoxic chemotherapy regimens, concomitant medications, and underlying liver comorbidities, may also have contributed to this finding.

In the literature, however, ALT normalization rates are reported to be similar among patients using ETV, TDF, and TAF [21,23]. In our study, no normalization of ALT was observed at the end of one year in any patient who had high ALT at baseline. The higher baseline ALT values in the tenofovir group and the lack of a significant decrease during follow-up may be related to the clinical characteristics of the patients in that group. This finding suggests that the difference between the groups might depend not only on the antiviral agents but also on the patients’ initial liver function status. The decrease in ALT levels in the ETV group from baseline to 6 months achieved statistical significance; however, the extent of this reduction was small and should be interpreted with caution in terms of clinical relevance.

Additionally, factors such as intensive chemotherapy administered to most patients with hematologic and oncologic malignancies, along with concurrent hepatotoxic drugs, malnutrition, and comorbid liver diseases, may have contributed to the lack of sustained ALT normalization. Given that our study was a single-center analysis with a limited sample size and heterogeneous patient population, these findings should be interpreted with caution and validated in larger multi-center cohorts. Our results also suggest that the biochemical response dynamics in immunosuppressed patients may differ from those in the general chronic hepatitis B population, and that antiviral efficacy should be assessed over longer follow-up periods.

One of the most notable findings of our study is that only one of 199 patients receiving prophylaxis developed HBV reactivation. This reactivation occurred after the patient voluntarily discontinued treatment, underscoring both the effectiveness of antiviral prophylaxis and the critical importance of adherence. Current clinical guidelines recommend that all patients scheduled for immunosuppressive therapy undergo HBV screening and initiate prophylaxis when indicated, and the widespread implementation of these measures plays a key role in preventing HBV reactivation.

## 5. Conclusions

This study examined the effectiveness of prophylactic antiviral treatments in preventing HBV reactivation in patients undergoing immunosuppressive therapy. According to our findings, only one patient who received prophylactic antiviral treatment developed HBV reactivation, and this occurred after they voluntarily discontinued the treatment. No reactivation was observed in any other patients. We also found that entecavir was the most frequently chosen antiviral agent. Its use was more common among the elderly population, likely due to concerns about the potential side effects of tenofovir. These results demonstrate that prophylactic antiviral treatments play a critical role in preventing HBV reactivation. Screening for HBV and starting prophylactic antiviral treatment when necessary can significantly reduce the risk of reactivation in patients scheduled for immunosuppressive therapy.

## Figures and Tables

**Table 1:** Characteristics of patients by HBV reactivation risk groups.

	Reactivation Risk			
	Low Risk	Moderate Risk	High Risk	*p*
	**(*n* = 82)**	**(*n* = 44)**	**(*n* = 73)**	
	***n*/(%)**	***n*/(%)**	***n*/(%)**	
**Age (Mean ± SD)**	61.3 ± 12.1	59.4 ± 12.6	60 ± 12.6	0.582
**Sex**				0.442
**Female**	44 (53.7)	23 (52.3)	32 (43.8)	
**Male**	38 (46.3)	21 (47.7)	41 (56.2)	
**HBsAg (+)**	9 (11)	14 (31.8)	30 (41.1)	**<0.001**
**Isolated anti-HBc (+)**	43 (52.4)	10 (22.7)	13 (17.8)	**<0.001**
**Anti-HBs (+) and anti-HBc (+)**	30 (36.6)	20 (45.5)	30 (41.1)	0.614
**HBV DNA detectable**	6 (7.3)	10 (22.7)	21(21.8)	**0.002**
**Baseline ALT levels**				0.746
**Mean ± SD**	22.5 ± 16.5	22.5 ± 10.8	22.3 ± 13.2	
**Median**	19.5	20.0	22.0	
**Antiviral treatment**				0.668
**ETV**	61 (74.4)	36 (81.8)	55 (75.3)	
**TAF**	13 (15.9)	5 (11.4)	8 (11.0)	
**TDF**	8 (9.8)	3 (6.8)	10 (13.7)	

Note: HBsAg: hepatitis B surface antigen; HBc: hepatitis B core antibody; HBs: hepatitis B surface antibody. Entecavir: ETV; Tenofovir alafenamide: TAF; Tenofovir disoproxil fumarate: TDF.

**Table 2:** Characteristics of Patients by Antiviral Treatment.

	ETV (*n* = 152)	TAF + TDF (*n* = 47)	*p*
	*n*/(%)	*n*/(%)	
**Age (Mean ± SD)**	61.5 ± 11.6	56.8 ± 14	**0.025**
**Sex**			0.382
**Female**	73 (48)	26 (55.3)	
**Male**	79 (52)	21 (44.7)	
**HBsAg (+)**	37 (24.3)	16 (34)	0.189
**Isolated anti-HBc (+)**	52 (34.2)	14 (29.8)	0.573
**Anti-HBs (+) and anti-HBc (+)**	41 (36.0)	12 (30.8)	0.568
**HBV DNA detectable**	24 (15.8)	13 (27.7)	0.068
**ALT (Mean ± SD)**			
**Baseline**	21.1 ± 12.5	27.1 ± 17.8	**0.022**
**6rt month**	19.5 ± 9.3	26.4 ± 17.3	**0.002**
**12th month**	20.7 ± 15.9	22.1 ± 8.0	**0.033**
**Baseline ALT**			**0.002**
**Normal**	135 (88.8)	33 (70.2)	
**High**	17 (11.2)	14 (29.8)	
**6th month ALT**			**0.006**
**Normal**	137 (90.1)	35 (74.5)	
**High**	15 (9.9)	12 (25.5)	
**12th month ALT**			**0.003**
**Normal**	135 (88.8)	33 (70.2)	
**High**	17 (11.2)	14 (29.8)	

Note: ALT: alanine aminotransferase (values classified according to the upper limit of normal, ULN = 35 U/L); HBsAg: hepatitis B surface antigen; anti-HBc: hepatitis B core antibody; anti-HBs: hepatitis B surface antibody; ETV: entecavir; TDF: tenofovir disoproxil fumarate; TAF: tenofovir alafenamide.

## Data Availability

The data presented in this study are available on request from the corresponding author. The data are not publicly available due to privacy and ethical restrictions.

## Abbreviations

HBV	Hepatitis B virus
HBsAg	Hepatitis B surface antigen
anti-HBc	Hepatitis B core antibody
anti-HBs	Hepatitis B surface antibody
ETV	Entecavir
TAF	Tenofovir alafenamide
TDF	Tenofovir disoproxil fumarate
AGA	American Gastroenterological Association
ALT	Alanine aminotransferase
EASL	European Association for the Study of the Liver
AASLD	American Association for the Study of Liver Diseases
